# Emotional Intelligence and Cognitive Flexibility as Predictors of Academic Success and Adaptation Outcomes Among International Students in Saudi Universities

**DOI:** 10.3390/jintelligence14050088

**Published:** 2026-05-19

**Authors:** Mubarak S. Aldosari, Haroon N. Alsager

**Affiliations:** 1 Special Education Department, College of Education, Prince Sattam bin Abdulaziz University, Al-Kharj 16273, Saudi Arabia; 2 Department of English, College of Science and Humanities, Prince Sattam bin Abdulaziz University, Al-Kharj 16273, Saudi Arabia; h.alsager@psau.edu.sa

**Keywords:** emotional intelligence, cognitive flexibility, international students, academic success, sociocultural adaptation, Saudi Arabia

## Abstract

International students in Saudi universities face academic and adaptation challenges shaped by emotional, cognitive, linguistic, and sociocultural factors. This study examined whether emotional intelligence and cognitive flexibility predicted academic success and adaptation outcomes among international students in Saudi public universities. A quantitative cross-sectional survey was conducted with 410 international students using structured measures of emotional intelligence, cognitive flexibility, academic success, adaptation outcomes, Arabic proficiency, and sociodemographic characteristics. Data were analysed using descriptive statistics, chi-square tests, Kendall’s tau-b correlations, hierarchical regression, and observed-variable path analysis. Duration of residence was significantly associated with Arabic proficiency, χ^2^(8) = 82.40, *p* < .001. Arabic proficiency was positively associated with GPA, τ = 0.62, *p* < .001, and adaptation outcomes, τ = 0.48, *p* < .001. In hierarchical regression, sociocultural covariates and psychological predictors explained substantial variance in academic success, R^2^ = 0.53, and adaptation outcomes, R^2^ = 0.53. Emotional intelligence and cognitive flexibility remained positive predictors of both outcomes after accounting for Arabic proficiency, duration of residence, region of origin, and language of instruction. Findings suggest that international student success in Saudi universities reflects an interaction of emotional, cognitive, linguistic, and contextual resources. Universities should strengthen integrated support for emotional regulation, adaptive thinking, Arabic-language development, and culturally responsive academic guidance.

## 1. Introduction

The internationalization of higher education has become a defining feature of contemporary universities, with student mobility serving as both an academic and geopolitical strategy (Hsieh, 2020). According to UNESCO (2023), the number of internationally mobile students has more than doubled over the past two decades, showing the need for host countries to understand not only academic outcomes but also the psychosocial processes that underpin successful adaptation. Saudi Arabia, through Vision 2030, has made deliberate investments in positioning itself as a regional and global hub for higher education by attracting international students across diverse disciplines and cultural backgrounds (Mohiuddin et al., 2023). International students studying in Saudi Arabia encounter a distinct educational and sociocultural environment. Differences in language of instruction, pedagogical styles, assessment practices, and institutional expectations may require substantial adjustment (DeLuca et al., 2021). In addition, broader sociocultural norms, religious practices, and communication patterns can differ markedly from those in students’ home countries. While many international students successfully navigate these transitions, others experience academic stress, performance difficulties, and challenges in adaptation (Ansari Lari et al., 2025; Wu et al., 2024). These differences suggest that, beyond cognitive ability alone, individual psychological resources may play a crucial role in determining academic success and overall adjustment within the Saudi higher education system.

Academic success has traditionally been conceptualized in terms of measurable performance indicators such as grade point average (GPA), task completion, and academic persistence (Cao et al., 2024). However, contemporary educational research emphasizes that academic outcomes are influenced not only by intellectual capacity but also by emotional regulation, adaptability, and self-regulatory competencies. For international students, the ability to manage stress, interpret social cues, and adjust cognitive strategies in response to unfamiliar academic norms may be particularly critical (Kristiana et al., 2022). At the same time, academic success cannot be fully understood without considering broader adaptation outcomes (Cárdenas et al., 2022). Adaptation outcomes refer to the extent to which students effectively adjust to their host university environment, including their comfort in social interactions, perceived competence in navigating institutional expectations, and overall adjustment to life in the host country (Han et al., 2022). In culturally distinct systems such as Saudi Arabia’s, academic success and adaptation outcomes are likely intertwined yet analytically distinct dimensions of student functioning.

Despite this growth, research examining international students’ experiences in Saudi Arabia remains underdeveloped. Much of the existing international student literature is grounded in Western host contexts, which limits the generalizability of prevailing adaptation models to culturally distinct environments such as Saudi Arabia. The Saudi context is characterized by unique sociocultural, religious, and linguistic norms that may pose distinct cognitive and emotional demands on international students (Alasmari, 2023). Consequently, there is a critical need for theory-driven research that examines how individual-level psychological resources enable students to navigate these demands effectively (Sarwer et al., 2025). Recent scholarship on higher education reform in Saudi Arabia has emphasized the need to move beyond recall-based assessment toward the development of higher-order cognitive and adaptive competencies aligned with Vision 2030 (Aldosari, 2025). As assessment practices shift toward promoting analytical thinking, resilience, and conceptual understanding, students are increasingly required to demonstrate not only subject mastery but also emotional regulation and cognitive adaptability (Salem et al., 2025; McGuire, 2025). In theory-based programs particularly, success depends on students’ ability to manage academic stress, engage in flexible problem-solving, and respond constructively to evaluative feedback (Blindheim et al., 2025; Chongjin et al., 2025). Within this evolving educational landscape, emotional intelligence and cognitive flexibility become especially salient psychological resources. Examining student’s predictive role in academic success and adaptation outcomes is therefore consistent with ongoing national reforms aimed at strengthening student competencies and enhancing the effectiveness of Saudi higher education institutions.

Recent advances in cross-cultural psychology suggest that cognitive and emotional capacities, rather than demographic characteristics alone, play a central role in determining adaptation outcomes (Carriedo et al., 2024). Beyond academic performance, international students must also achieve broader adaptation outcomes to function effectively within a new educational and cultural environment (Attrill et al., 2016). Adaptation outcomes refer to the extent to which students adjust psychologically, socially, and behaviorally to the demands of the host university context (Al Juboori et al., 2025). In culturally distinct higher education systems such as Saudi Arabia, where linguistic, pedagogical, and social norms may differ substantially from students’ home contexts, adaptation outcomes represent a critical dimension of overall student functioning. Therefore, examining both academic success and adaptation outcomes provides a more comprehensive understanding of how emotional intelligence and cognitive flexibility contribute to international students’ effectiveness and adjustment in Saudi universities.

Despite growing research on international student adjustment, several important gaps remain in the literature. First, much of the existing evidence examining emotional intelligence, cognitive flexibility, and student adaptation has been conducted in Western educational contexts, limiting understanding of how these psychological resources operate within Middle Eastern higher education systems. Second, previous studies have often examined academic success or adaptation independently rather than considering both outcomes simultaneously. Third, limited research has explored the combined predictive roles of emotional intelligence and cognitive flexibility among international students studying in culturally and linguistically distinct educational environments such as Saudi Arabia. This limitation is particularly important given the rapid internationalization of Saudi higher education under Vision 2030 and the substantial growth of government-sponsored international student enrollment across Saudi universities. This study, therefore, aims to investigate the predictive ability of emotional intelligence and cognitive flexibility in explaining both academic success and adaptation outcomes among international students enrolled in Saudi universities. By examining performance-based indicators alongside broader adjustment outcomes, the study adopts a more comprehensive view of student effectiveness within a culturally distinct higher education context. Grounded in established theoretical frameworks and employing validated psychometric instruments, the research seeks to generate findings that are both contextually relevant and theoretically generalizable.

### Theoretical Framework

The present study is grounded in an integrative theoretical framework that combines cultural adaptation theory with contemporary models of emotional intelligence, cognitive flexibility, and academic performance to explain both academic success and broader adaptation outcomes among international students in Saudi universities. Rather than treating academic performance and adaptation as isolated processes, the framework conceptualizes them as interrelated outcomes shaped by underlying psychological resources.

Cultural adaptation theory (Ward et al., 2001) provides the contextual foundation for this study. The theory distinguishes between psychological adaptation, which reflects the behavioral competence required to manage daily interactions, institutional expectations, and cultural norms in the host society. In international higher education contexts, adaptation extends beyond social functioning to include the ability to navigate unfamiliar academic systems, instructional styles, and assessment standards. For students studying in culturally distinct environments such as Saudi Arabia, adaptation requires not only intellectual competence but also emotional resilience and cognitive openness. Thus, adaptation outcomes are conceptualized in this study as functional indicators of students’ ability to operate effectively within both academic and sociocultural dimensions of the host university environment.

Emotional intelligence theory (Mayer et al., 2008) contributes a mechanism-based explanation for how individuals manage the emotional demands inherent in cross-cultural academic settings. Emotional intelligence encompasses the abilities to perceive, understand, regulate, and utilize emotions constructively. Within international education contexts, students are frequently exposed to stressors such as language barriers, performance pressures, social ambiguity, and cultural misunderstandings. Students with higher emotional intelligence are theorized to regulate anxiety, maintain motivation, interpret interpersonal cues accurately, and sustain adaptive coping strategies. These emotional regulation capacities not only support psychological well-being but also enhance academic persistence, classroom engagement, and effective communication with peers and instructors. Consequently, emotional intelligence is positioned as a foundational psychological resource contributing to both academic success and broader adaptation outcomes.

Complementing this emotional regulation perspective, cognitive flexibility theory (Martin & Rubin, 1995) provides a cognitive adaptation mechanism. Cognitive flexibility refers to an individual’s capacity to shift perspectives, generate alternative solutions, reinterpret challenges, and modify thinking strategies in response to changing demands. In culturally unfamiliar educational systems, rigid cognitive schemas may hinder students’ ability to adjust to new pedagogical approaches, assessment methods, or communication styles. By contrast, cognitively flexible students are more likely to reinterpret academic challenges constructively, integrate new cultural information, and adjust problem-solving strategies effectively. This cognitive adaptability reduces maladaptive rigidity and facilitates smoother adjustment across both academic and social domains. Thus, cognitive flexibility is conceptualized as a cognitive resource that supports effective adaptation and sustained academic functioning.

Emotional intelligence and cognitive flexibility are theoretically interconnected psychological resources that may jointly facilitate successful academic and sociocultural functioning. Emotional intelligence primarily supports emotional regulation, stress management, and interpersonal responsiveness, whereas cognitive flexibility enables individuals to reinterpret unfamiliar situations, shift perspectives, and adapt cognitive strategies. Within culturally unfamiliar educational environments, these capacities may operate synergistically, such that emotionally intelligent students are better able to regulate emotional responses to adaptation challenges, while cognitively flexible students are better able to modify behavioral and cognitive responses to those challenges. Together, these competencies may enhance both academic functioning and broader adaptation outcomes among international students.

Finally, contemporary models of academic success emphasize the role of self-regulation, motivational control, and adaptive psychological functioning in predicting academic outcomes beyond intellectual ability alone. Academic performance is increasingly understood as the product of cognitive, emotional, and behavioral self-management processes. Emotional intelligence enhances self-regulatory capacity under stress, while cognitive flexibility promotes adaptive learning strategies and responsiveness to feedback. Together, these psychological competencies contribute to improved academic achievement, sustained engagement, and effective adaptation within complex educational environments.

By synthesizing these theoretical perspectives, the present framework proposes that emotional intelligence and cognitive flexibility function as core psychological resources that enhance international students’ capacity to achieve academic success while simultaneously facilitating adaptation to the academic and sociocultural demands of Saudi universities. This integrated model provides a coherent explanation of how emotional regulation and cognitive adaptability jointly contribute to performance and adjustment outcomes in culturally diverse higher education settings.

Thus, this study examines the predictive ability of emotional intelligence and cognitive flexibility in explaining both academic success and adaptation outcomes among international students enrolled in Saudi universities. Specifically, the study seeks to determine the levels of emotional intelligence, cognitive flexibility, and academic success among international students, as well as to evaluate the extent to which emotional intelligence and cognitive flexibility predict academic success. In addition, the study aims to assess the predictive power of emotional intelligence and cognitive flexibility on students’ adaptation outcomes within the academic and sociocultural environment of Saudi universities.

## 2. Materials and Methods

This study employed a quantitative cross-sectional survey to examine the predictive roles of emotional intelligence and cognitive flexibility in relation to academic success and adaptation outcomes among international students enrolled in Saudi universities. According to official statistics from the Ministry of Education, approximately 98,453 international students are currently studying in public and private universities across the Kingdom, providing a substantial population for investigating psychological factors associated with academic functioning and adaptation in the Saudi higher education context.

### 2.1. Survey Instrument and Study Design

Data were collected using a structured self-administered questionnaire developed from validated instruments widely used in educational and psychological research. The questionnaire was designed to measure four key constructs: emotional intelligence, cognitive flexibility, academic success, and adaptation outcomes. Adaptation outcome items were adapted from prior sociocultural adjustment and international student adaptation literature and contextualized to the Saudi higher education environment. Items assessing emotional intelligence were adapted from the Wong and Law Emotional Intelligence Scale (WLEIS), while cognitive flexibility items were derived from established cognitive flexibility measures such as emotional intelligence and cognitive flexibility (Traymbak et al., 2022). Additional items were included to capture students’ perceived academic success and broader adaptation outcomes within the host educational environment. Prior to full administration, the questionnaire underwent a pilot testing phase to assess clarity, reliability, and construct validity. A pilot sample of international students was recruited to complete the preliminary version of the instrument. Feedback from this stage was used to refine wording, remove ambiguous items, and ensure cultural and linguistic appropriateness. Internal consistency reliability of the scales was assessed using Cronbach’s alpha coefficients, with all constructs demonstrating acceptable reliability thresholds prior to the main survey administration.

The final questionnaire consisted of five sections. The first section collected demographic information, including gender, nationality, region of origin, program level, field of study, language of instruction, length of stay in Saudi Arabia, Arabic proficiency, and current grade point average (GPA). The second section measured emotional intelligence using 16 items. The third section assessed cognitive flexibility through 6 items. The fourth section measured academic success using 5 items reflecting students’ perceived academic competence and performance. The final section measured adaptation outcomes using 5 items related to students’ adjustment to the academic and social environment of Saudi universities.

All attitudinal items were measured using a five-point Likert scale ranging from 1 (strongly disagree) to 5 (strongly agree). This scale was selected to allow respondents to express varying degrees of agreement while maintaining analytical consistency across constructs. The structured design of the instrument enabled standardized data collection and facilitated subsequent statistical analysis of the relationships between emotional intelligence, cognitive flexibility, academic success, and adaptation outcomes.

### 2.2. Sampling and Data Collection

Eligibility criteria were designed to ensure that participants had sufficient exposure to the Saudi higher education environment to meaningfully evaluate their academic experiences and adaptation processes. International students enrolled in Saudi universities represent a diverse yet distinctive population shaped by national scholarship policies and program structures. The majority of international students studying in Saudi Arabia are admitted through fully funded government scholarship programs. These scholarships are primarily concentrated in Arabic language and Islamic studies programs, which attract students from a wide range of countries. These programs are delivered predominantly in Arabic, reflecting their disciplinary and cultural foundations. In contrast, programs in science, engineering, and other technical fields are typically taught in English. It is also important to note that Saudi universities generally do not offer full scholarships to international students in medical programs, which further shapes the distribution of international student enrollment across academic disciplines.

The sampling frame for this study was informed by the most recent statistics published by the Saudi Ministry of Education, which report approximately 98,453 international students enrolled in public and private universities across the Kingdom. Based on population size, a post hoc statistical power calculation was conducted for the main multiple regression analyses using an alpha level of 0.05, a sample size of 400, and a medium effect size of *f*^2^ = 0.15. Based on these parameters, the achieved statistical power exceeded 0.99 for detecting significant predictive effects in the regression models. This indicates that the sample size was adequate for testing the predictive contributions of emotional intelligence, cognitive flexibility, and the included sociocultural covariates on academic success and adaptation outcomes. Therefore, the final sample of 400 participants provided sufficient statistical power for the planned analyses. This sample size also accommodates the analytical requirements of multivariate statistical techniques, including multiple regression and structural equation modeling, while accounting for potential non-response or incomplete questionnaires. Moreover, a sample of this size allows for exploratory subgroup comparisons based on demographic characteristics such as academic level and region of origin.

Participants were recruited through collaboration with international student offices, academic departments, and student organizations within Saudi universities. Recruitment notices clearly explained the purpose of the study, emphasized voluntary participation, and assured respondents of confidentiality and anonymity. Invitations to participate were disseminated through institutional email lists, university communication platforms, and social media channels commonly used by international student communities. Given the linguistic diversity of the international student population, they were giving the option to choose the survey in Arabic or English. This approach helped maintain measurement consistency while ensuring that participants from diverse linguistic backgrounds could respond accurately and comfortably.

### 2.3. Participant Design

Participants consist of international students enrolled in accredited public universities across Saudi Arabia. The definition of “international student” for this study includes any student who is not a citizen of the Kingdom and who has relocated to Saudi Arabia for the primary purpose of pursuing higher education. Eligibility criteria are intentionally broad to capture a wide range of academic disciplines, linguistic backgrounds, and cultural contexts, which strengthens the external validity of the findings.

### 2.4. Participants Must Meet the Following Inclusion Criteria

#### 2.4.1. Non-Saudi Nationality

Currently enrolled in a bachelor’s, master’s, or doctoral program in a Saudi university.

#### 2.4.2. Minimum Residency of One Academic Semester or More

These criteria help ensure that students have had sufficient exposure to the host educational environment to form meaningful perceptions of adaptation and academic engagement.

### 2.5. Sampling Strategy

This study uses a stratified random sampling strategy to enhance representativeness. The sampling frame was stratified by academic level (undergraduate vs. postgraduate) and the region of origin. Stratification ensures the sample reflects key demographic and academic diversity within the population of international students. Within each stratum, participants will be randomly selected from institutional registries where available or through systematic outreach strategies coordinated with international student offices.

### 2.6. Data Collection Procedure

Data was collected using an online survey platform. This approach ensures accessibility across geographic locations and accommodates international students who may be on campus, online, or in off-campus accommodations. The instrument was structured into sections for demographics, emotional intelligence, cognitive flexibility, academic success, and adaptation outcomes. Before distribution, institutional review board (IRB) approval and Ministry of Education clearance were confirmed. Recruitment messages included a brief overview of the research, expected completion time (approximately 15–20 min), assurance of voluntary participation, confidentiality and anonymity safeguards, and contact information for the principal investigator and ethics office. The survey opened with an informed consent form, which participants must electronically agree to before proceeding. The consent form outlines the purpose, procedures, risks (if any), benefits, and confidentiality protections. Non-response follow-up reminders were sent at one and two weeks after the initial invitation, consistent with best practices for increasing response rates in online surveys. Data collection remained open for 4–6 weeks to maximize participation.

### 2.7. Ethical Clearance and Ethics Employed

This study received formal ethical approval from the Kingdom of Saudi Arabia, Ministry of Education and Prince Sattam bin Abdulaziz University under approval number SCBR-655/2026. Ethical principles guiding this research adhere to internationally recognized standards for research involving human subjects, including respect for persons, beneficence, and justice.

### 2.8. Data Management and Analysis

After data collection, responses were downloaded in a secure format (CSV/XLSX) and processed in statistical software (IBM SPSS Statistics v31). Internal consistency reliability was assessed using both Cronbach’s alpha and McDonald’s omega. Cronbach’s alpha was reported because it is widely used in educational and psychological research, while McDonald’s omega was included because it provides a complementary reliability estimate that is less dependent on the assumption of equal item loadings. Reliability was examined for the overall scales and their sub-dimensions using the raw item-level questionnaire responses. Internal consistency was examined using Cronbach’s alpha and McDonald’s omega based on the raw item-level questionnaire responses. For emotional intelligence, the overall WLEIS demonstrated good reliability, with Cronbach’s alpha = 0.82 and McDonald’s omega = 0.82. At the dimensional level, self-emotion appraisal showed α = 0.59 and ω = 0.58, others’ emotion appraisal showed α = 0.63 and ω = 0.67, use of emotion showed α = 0.74 and ω = 0.75, and regulation of emotion showed α = 0.49 and ω = 0.58. These results indicate that the overall emotional intelligence scale had adequate internal consistency, although some sub-dimensions showed weaker reliability and should therefore be interpreted with caution. For professional compatibility, reliability varies across the PPEFS dimensions. Person–organization fit demonstrated strong reliability, with α = 0.84 and ω = 0.87, while person–group fit showed acceptable reliability, with α = 0.77 and ω = 0.73. Person–supervisor fit showed marginal-to-acceptable reliability, with α = 0.68 and ω = 0.71. However, person–job fit demonstrated poor internal consistency, with α = −0.24 and ω = 0.18. The overall professional compatibility scale also showed low internal consistency, with α = 0.39 and ω = 0.00. These findings suggest that professional compatibility should be interpreted primarily at the subscale level rather than as a single total score, particularly because the PPEFS captures multiple theoretically distinct forms of fit, including job, organization, group, and supervisor fit. Descriptive statistics (means, standard deviations, frequencies) were calculated for all study variables. Pearson correlation coefficients were computed to examine bivariate associations among EI, CF, academic success, and sociocultural adaptation indicators. Statistical significance was assessed at a two-sided α level of 0.05.

Prior to the main analyses, the psychometric properties of the study measures were evaluated to ensure reliability and construct adequacy within the study sample. Internal consistency reliability was assessed using Cronbach’s alpha coefficients, and all study constructs demonstrated acceptable reliability levels exceeding recommended thresholds. Confirmatory factor analysis (CFA) was initially conducted to evaluate the adequacy of the proposed measurement structure. The CFA results generally supported the construct structure of the principal measures and indicated acceptable model fit across standard fit indices. However, during latent structural estimation, instability emerged within the full latent model, including covariance estimation difficulties and reduced model stability associated with sample heterogeneity and overlapping construct indicators. To improve estimation stability and model parsimony, validated composite scores were subsequently computed and used in the final observed-variable path analysis. Accordingly, the final predictive model was estimated as a path analysis using observed composite variables rather than a full latent structural equation model. Data screening procedures were conducted prior to analysis, including assessment of missing data, normality, outliers, and multicollinearity assumptions. Missing data were minimal and did not substantially affect the analyses. Multicollinearity diagnostics using variance inflation factor (VIF) and tolerance statistics indicated acceptable levels across predictors. Given the ordinal nature of Arabic proficiency, additional non-parametric correlations using Kendall’s tau-b were conducted to confirm the robustness of associations involving ordinal variables. Hierarchical multiple regression analyses were then performed to examine the predictive contributions of emotional intelligence and cognitive flexibility beyond demographic and sociocultural covariates, including Arabic proficiency, duration of residence, region of origin, and language of instruction.

Confirmatory factor analysis (CFA) was initially conducted to evaluate the factorial validity of the study constructs. The CFA results generally supported the proposed factor structure and demonstrated acceptable construct validity across the principal measures. However, during structural model estimation, instability emerged within the latent variable specification, including covariance estimation difficulties and reduced model stability associated with sample heterogeneity and overlapping construct indicators. To improve model parsimony and estimation stability, composite observed scores were subsequently computed for each validated construct and used in the final predictive path analysis. Accordingly, the final structural model was estimated as an observed-variable path analysis rather than a full latent structural equation model.

Construct validity was examined via CFA using maximum likelihood estimation. The hypothesised four-factor EI model and one-factor CF model were tested separately. Model fit was evaluated using multiple indices: comparative fit index (CFI), Tucker–Lewis index (TLI), root mean square error of approximation (RMSEA), and standardised root mean square residual (SRMR). Conventional cut-offs (CFI/TLI ≥ 0.90; RMSEA ≤ 0.08; SRMR ≤ 0.08) were used as reference thresholds, while recognising the limitations of strict cut-off reliance. Measurement invariance across key demographic groups was explored by comparing model fit across subgroups using multi-group CFA procedures. Configural stability was examined first, followed by constrained models where feasible.

Hierarchical multiple regression analyses were conducted to examine the predictive contributions of emotional intelligence and cognitive flexibility beyond demographic and sociocultural covariates. In the first block, Arabic proficiency, duration of residence, region of origin, and language of instruction were entered as control variables. Emotional intelligence and cognitive flexibility were entered in the second block to assess their incremental predictive effects on academic success and adaptation outcomes. Given the ordinal nature of Arabic proficiency and duration of residence categories, Kendall’s tau-b correlations were additionally computed to verify the robustness of associations involving ordinal variables. Multicollinearity diagnostics were assessed using variance inflation factor (VIF) and tolerance statistics prior to regression analysis. Multicollinearity was assessed using variance inflation factor (VIF) and tolerance statistics prior to regression analysis.

Structural equation modeling (SEM) was employed to examine simultaneous relationships among EI, CF, and academic and sociocultural adaptation outcomes (Objective 3). Observed composite scores were used as indicators in structural models due to instability in latent measurement structures identified in CFA. Standardised path coefficients were estimated using maximum likelihood procedures. Model interpretation focused primarily on structural path estimates given near-saturated model conditions. Mediation Analysis was used to assess whether sociocultural adaptation mediated the relationship between emotional intelligence and academic success, indirect effects were estimated using non-parametric bootstrapping with 5000 resamples. Bias-corrected 95% confidence intervals were generated. Mediation was considered statistically significant if the bootstrapped confidence interval did not include zero. All hypothesis tests were two-sided with statistical significance defined as *p* < 0.05. Effect sizes, confidence intervals, and standardised coefficients are reported to facilitate interpretation. No formal correction for multiple comparisons was applied, as analyses were hypothesis-driven and aligned with prespecified research objectives.

Software management: Data management, descriptive statistics, reliability analysis, correlation analysis, and hierarchical regression analyses were conducted using IBM SPSS Statistics version 30.0 (IBM Corp., Armonk, NY, USA). Confirmatory factor analysis (CFA) and path analysis were performed using IBM SPSS AMOS version 30.0. Additional assumption testing and collinearity diagnostics were conducted prior to model estimation.

## 3. Results

Table 1 shows the sociodemographic characteristics of the 410 international students who participated in the study. The sample comprised 260 males (63.4%) and 150 females (36.6%). Participants originated primarily from the Middle East and North Africa (MENA) (43.4%) and Sub-Saharan Africa (30.0%), with smaller representations from South Asia (10.5%), East/Southeast Asia (5.6%), Europe (6.8%), and other regions (3.7%). Most participants were enrolled in graduate programmes (65.1%), while 34.9% were undergraduates. Arabic was the primary language of instruction for 51.5% of students, English for 41.2%, and mixed instruction for 7.3%. Participants had resided in Saudi Arabia for an average of 19.8 months (SD = 11.6). Approximately 56.1% had lived in the country for 1–2 years. Arabic proficiency was distributed across levels, with the largest proportion reporting moderate proficiency (32.7%), followed by good proficiency (30.0%). The mean GPA (5-point scale) was 3.64 (SD = 0.65), indicating generally high academic performance.

Table 2 highlights notable variation in Arabic proficiency across demographic and academic characteristics. Overall, Arabic proficiency was concentrated mainly at the moderate and good levels. Among males, the largest proportion reported moderate proficiency (100/260, 38.5%), whereas among females the largest proportion reported good proficiency (46/150, 30.7%). However, females also had a higher proportion reporting very low proficiency than males (24.0% vs. 15.4%).

Language of instruction differed by programme level. Undergraduate students were predominantly taught in Arabic (89/143, 62.2%), while graduate students were almost equally distributed between English (120/267, 44.9%) and Arabic (122/267, 45.7%). Mixed-language instruction was more frequent among graduate students than undergraduates (9.4% vs. 3.5%).

By region of origin, respondents from the MENA region formed the largest group and were mainly distributed across moderate and good proficiency levels. Sub-Saharan African respondents also showed a similar concentration in the moderate and good categories. Duration of stay showed the clearest pattern: respondents who had lived in Saudi Arabia for more than 2 years were predominantly in the good proficiency category (60/95), whereas those with shorter stays were more often classified as moderate.

Significant associations were observed between several structural variables. Gender was associated with Arabic proficiency, χ^2^(4) = 13.27, *p* = .010, Cramér’s V = 0.18, indicating a small-to-moderate effect (Table 3). Males were more frequently represented in the moderate proficiency category, whereas females were slightly overrepresented in the very low proficiency category. Programme level was significantly associated with language of instruction, χ^2^(2) = 11.88, *p* = .003, V = 0.17. Undergraduates were more likely to be enrolled in Arabic-taught programmes compared with graduates. Region of origin was also associated with Arabic proficiency, χ^2^(20) = 46.20, *p* < .001, V = 0.17, suggesting that linguistic integration varies across regional backgrounds. The strongest structural association was observed between duration of residence and Arabic proficiency, χ^2^(8) = 82.40, *p* < .001, V = 0.32. Students residing in Saudi Arabia for more than two years were substantially more likely to report good proficiency levels. This moderate effect size indicates that length of exposure is a central factor in sociocultural adaptation. Given the ordinal nature of Arabic proficiency, additional non-parametric correlations using Kendall’s tau-b were conducted to verify the robustness of the associations involving ordinal variables. Results indicated that Arabic proficiency remained positively associated with GPA (τ = 0.62, *p* < .001) and adaptation outcomes (τ = 0.48, *p* < .001), supporting the consistency of the findings obtained through parametric analyses.

Table 4 presents the correlations among the principal study variables. Emotional intelligence demonstrated significant positive associations with cognitive flexibility, academic success, adaptation outcomes, Arabic proficiency, and duration of residence. Cognitive flexibility was also positively associated with academic success and adaptation outcomes. Arabic proficiency showed a particularly strong positive relationship with academic success and adaptation outcomes, suggesting that language competence may play an important role in students’ academic and sociocultural functioning within Saudi universities. Given the ordinal nature of Arabic proficiency and duration of residence, additional non-parametric correlations using Kendall’s tau-b were conducted to verify the robustness of the observed relationships. The results remained consistent with the parametric analyses, supporting the stability of the reported associations.

Hierarchical multiple regression analysis was conducted to examine the predictive contributions of emotional intelligence and cognitive flexibility on academic success after controlling for sociocultural and demographic variables (Table 5). In Block 1, Arabic proficiency, duration of residence, region of origin, and language of instruction were entered as covariates. This model explained 39% of the variance in academic success. In Block 2, emotional intelligence and cognitive flexibility were entered as psychological predictors. The inclusion of these variables significantly improved the model, explaining an additional 14% of variance in academic success. Emotional intelligence emerged as the strongest psychological predictor of academic success, while cognitive flexibility also demonstrated a significant positive effect. Collinearity diagnostics indicated acceptable levels of multicollinearity across predictors, with VIF values below the recommended threshold of 5.

A second hierarchical multiple regression analysis was conducted to examine predictors of adaptation outcomes (Table 6). Sociocultural variables entered in Block 1 significantly predicted adaptation outcomes, particularly Arabic proficiency and duration of residence. The inclusion of emotional intelligence and cognitive flexibility in Block 2 significantly improved the explanatory power of the model. Emotional intelligence demonstrated the strongest positive association with adaptation outcomes, suggesting that students with greater emotional regulation and interpersonal competence reported better adjustment to the Saudi university environment. Cognitive flexibility also significantly predicted adaptation outcomes, indicating that adaptive thinking and perspective-shifting abilities may facilitate sociocultural and academic adjustment. All predictors demonstrated acceptable collinearity statistics, with VIF values remaining within recommended limits.

**Figure 1:**
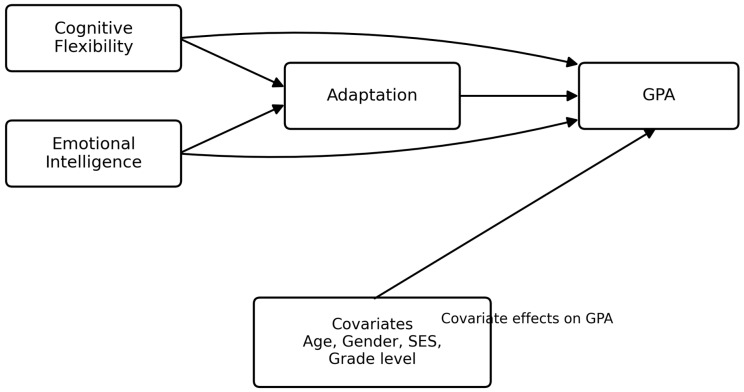
Observed-variable path model predicting academic success and adaptation outcomes among international students in Saudi universities.

In Table 7, a structural path model was estimated to examine direct associations between emotional intelligence, cognitive flexibility, adaptation (proxied by Arabic proficiency), and academic performance. Emotional intelligence was positively associated with adaptation (β = 0.21, *p* < .001) and directly associated with GPA (β = 0.16, *p* = .002). Cognitive flexibility was positively associated with adaptation (β = 0.18, *p* = .004) but negatively associated with GPA in the fully specified model (β = −0.27, *p* = .001). Given the limited degrees of freedom in the structural specification, model fit indices were not substantively informative. Accordingly, results should be interpreted as structural associations rather than confirmatory evidence of model fit. To facilitate interpretation of the analytical model, the observed-variable path model tested in this study is presented in Figure 1.

In Table 8, a bootstrapped indirect effects analysis (5000 resamples) examined whether adaptation (Arabic proficiency) statistically accounted for the association between emotional intelligence and academic performance. The indirect effect was statistically significant (indirect β = 0.026, 95% CI: 0.012–0.041, *p* = .001), suggesting that part of the association between emotional intelligence and GPA operates through adaptation. Given the cross-sectional design, this finding should be interpreted as evidence of statistical mediation rather than causal mediation.

## 4. Discussion

This study examined the role of emotional intelligence and cognitive flexibility in predicting academic success and sociocultural adaptation among international students studying in Saudi Arabia. By integrating psychological and contextual variables, the analysis aimed to provide a more comprehensive understanding of the factors that shape international students’ academic experiences. The present findings indicate that emotional intelligence and cognitive flexibility significantly predicted both academic success and adaptation outcomes even after controlling for sociocultural variables such as Arabic proficiency, duration of residence, and regional background. This suggests that emotional and cognitive psychological resources contribute uniquely to international students’ functioning beyond structural and linguistic adjustment factors. Further, duration of residence also demonstrated a positive relationship with adaptation outcomes, suggesting that prolonged exposure to the host educational and sociocultural environment may facilitate adjustment processes over time. Students with longer residence periods may gradually develop greater familiarity with institutional expectations, communication practices, and social norms, thereby enhancing both academic and sociocultural adaptation. The present study findings indicate that the majority of participants reported moderate to high levels of emotional intelligence and cognitive flexibility. These results align with previous research, suggesting that international students often develop adaptive psychological capacities as they navigate unfamiliar academic and cultural environments (Matsumoto et al., 2006; Xu & Chai, 2025). Emotional intelligence has been conceptualized as the ability to perceive, understand, regulate, and utilize emotions effectively in social interactions (Bru-Luna et al., 2021). Such competencies may help students manage the emotional demands associated with studying in a foreign environment, including cultural adjustment, language barriers, and academic pressures.

Similarly, cognitive flexibility is defined as the ability to shift perspectives and adapt thinking in response to changing situational demands, which has been linked to effective problem solving and resilience in complex environments (Uddin, 2021). International students often encounter diverse teaching styles, assessment methods, and cultural expectations, all of which require flexible cognitive strategies. The relatively high levels of cognitive flexibility observed in this sample may therefore reflect the adaptive capacities required to succeed in an international academic context (Cubeddu & Martini, 2025). The sociocultural profile of this study revealed important structural patterns. Participants were primarily drawn from the Middle East and North Africa and Sub-Saharan Africa, reflecting regional mobility trends in higher education. The majority of students were enrolled in graduate programmes, and Arabic was the primary language of instruction for more than half of the sample. These characteristics highlight the diversity of international student populations and show the importance of contextual factors in shaping adaptation and academic outcomes.

One of the most notable findings from our study was the strong association between duration of residence in Saudi Arabia and Arabic language proficiency. Arabic proficiency emerged as a significant predictor of both academic success and adaptation outcomes, highlighting the importance of linguistic competence within the Saudi higher education context. Given that a substantial proportion of international students in Saudi Arabia are enrolled in Arabic language and Islamic studies programs delivered in Arabic, language proficiency may facilitate classroom engagement, institutional communication, and sociocultural integration. This finding aligns with prior international student literature emphasizing language competence as a critical factor in academic adjustment and intercultural adaptation. This finding is consistent with sociocultural adaptation theory, which posits that increased exposure to the host environment facilitates language acquisition and cultural adjustment (Wei, 2025; Haikuo, 2025). Language proficiency is widely recognized as a key determinant of academic integration because it affects students’ ability to participate in lectures, engage with course materials, and interact with peers and instructors (Huang et al., 2025). The association between regional origin and language proficiency further illustrates the role of structural factors in adaptation. Students from regions with greater linguistic or cultural proximity to Arabic-speaking environments tended to report higher levels of proficiency. This pattern reflects broader findings in international education research, which emphasize that cultural distance can influence the pace and ease of adaptation (Gao et al., 2024). Students from culturally similar backgrounds often experience fewer barriers to communication and social integration (McCabe et al., 2024).

The correlational analyses revealed several important relationships among the key constructs. Emotional intelligence was positively associated with cognitive flexibility, suggesting that these psychological capacities may operate together as complementary resources for managing complex academic environments (Butcher et al., 2026). This finding aligns with previous studies showing that individuals with higher emotional intelligence tend to demonstrate greater cognitive adaptability and problem-solving capacity (Smith et al., 2022; Xuan, 2025). A particularly strong correlation was observed between Arabic proficiency and academic performance. This relationship shows the critical role of language competence in shaping educational outcomes. Language proficiency not only influences comprehension of course content but also affects students’ confidence, participation, and sense of belonging within the academic community (Zhou & Li, 2025). The strong association observed in this study therefore reinforces the importance of linguistic adaptation as a key mechanism through which international students navigate academic challenges.

Further, the multiple regression analysis provided further insight into the predictors of academic success. Emotional intelligence emerged as a significant positive predictor of GPA, even after controlling for other variables. This finding is consistent with a growing body of literature suggesting that emotional competencies contribute to academic performance by supporting effective stress management, motivation, and interpersonal relationships (Shengyao et al., 2024). Students with higher emotional intelligence may be better equipped to cope with academic setbacks and maintain engagement with their studies. Interestingly, cognitive flexibility demonstrated a negative coefficient in the fully adjusted model despite showing a positive correlation with GPA at the bivariate level. This pattern suggests the presence of a statistical suppression effect, whereby the inclusion of highly correlated predictors alters the direction of the coefficient. Suppression effects can occur when predictors share overlapping variance with the outcome variable (Watson et al., 2013). In this context, the strong influence of Arabic proficiency on academic performance may have altered the apparent relationship between cognitive flexibility and GPA. Such findings highlight the importance of interpreting regression coefficients within the broader context of multivariate relationships.

Arabic proficiency emerged as the strongest predictor of academic success in the regression model. This result reinforces the central role of language competence in international student achievement. The ability to understand lectures, read academic texts, and communicate effectively with instructors and peers is essential for success in higher education. Language barriers can limit students’ participation in classroom discussions and reduce their confidence in academic settings, thereby affecting overall performance (Azram et al., 2025). The structural path analysis further examined the relationships among emotional intelligence, cognitive flexibility, adaptation, and academic performance. Emotional intelligence was positively associated with adaptation, indicating that students with stronger emotional competencies may find it easier to adjust to new sociocultural environments. Emotional regulation skills may enable students to manage the stress associated with cultural transition, maintain positive social interactions, and develop supportive networks within the host institution.

Cognitive flexibility also demonstrated a positive association with adaptation, suggesting that flexible thinking supports the ability to adjust to unfamiliar cultural norms and academic expectations. These findings align with previous research indicating that psychological adaptability plays a crucial role in successful intercultural adjustment (Ye & Dong, 2021). The mediation analysis suggested that adaptation partially accounted for the association between emotional intelligence and academic performance. Although the indirect effect was statistically significant, it is important to interpret this finding cautiously because the data were cross-sectional. While the results are consistent with theoretical models suggesting that psychological resources influence academic outcomes through adaptation processes, longitudinal studies would be needed to establish causal relationships. Collectively, the findings suggest that academic success and adaptation among international students are shaped by an interaction of emotional, cognitive, linguistic, and sociocultural factors rather than by psychological resources alone.

However, the findings should also be interpreted in light of the possibility of self-selection bias inherent in voluntary survey participation. International students who felt more comfortable discussing adaptation experiences or who possessed stronger linguistic and academic adjustment may have been more likely to participate in the study. Consequently, students experiencing more severe adaptation challenges or lower language proficiency may have been underrepresented. This consideration is particularly relevant within the Saudi higher education context, where international students demonstrate substantial diversity in linguistic competence, cultural familiarity, and educational background.

Finally, the findings of this study highlight the interconnected roles of psychological competencies and sociocultural adaptation in shaping academic success among international students. Emotional intelligence and cognitive flexibility appear to function as internal resources that help students navigate the challenges of studying in a foreign environment, while language proficiency serves as a key external factor that facilitates academic integration. These findings have important implications for higher education institutions that host international students. Universities may benefit from implementing programs that support both psychological and linguistic adaptation. For example, orientation programs, language support services, and intercultural training workshops can help students develop the skills needed to succeed academically and socially. In addition, interventions designed to enhance emotional intelligence, such as stress management training and peer mentoring programs, may contribute to improved academic outcomes.

### Limitations

Several limitations of this study should be acknowledged. First, cross-sectional design limits the ability to establish causal relationships among emotional intelligence, cognitive flexibility, academic success, and adaptation outcomes. The inclusion of sociocultural covariates improved explanatory precision; however, additional contextual variables such as institutional support, social integration, and financial adjustment were not examined and may also influence adaptation outcomes. Although the analyses identify significant associations between these variables, longitudinal research would be necessary to examine how psychological resources and adaptation processes develop over time among international students studying in Saudi Arabia.

Second, the study relied primarily on self-reported measures, which may introduce potential response biases such as social desirability or recall bias. Additionally, the voluntary nature of participation may have introduced self-selection bias, as students with greater confidence in their adaptation experiences or stronger language proficiency may have been more willing to complete the questionnaire. As a result, the perspectives of students experiencing more substantial adaptation difficulties may not have been fully captured. Although validated instruments were used to enhance measurement reliability and consistency, self-reported perceptions may not fully capture the complexity of students’ academic performance and adaptation experiences. Future studies may benefit from incorporating additional objective indicators or mixed method approaches to triangulate findings.

Third, the findings should be interpreted within the specific institutional and cultural context of Saudi higher education. The majority of international students in Saudi universities are enrolled through government-funded scholarship programs and are concentrated primarily in public universities rather than private institutions. Moreover, a substantial proportion of international students come to Saudi Arabia to study Arabic language and Islamic studies, reflecting the country’s role as a global center of Islamic scholarship and the linguistic significance of Arabic as the language of the Qur’an. As a result, the academic and cultural experiences of this population may differ from those of international students in other disciplinary fields or higher education systems.

These contextual characteristics may limit the broader generalizability of the findings to international student populations in other countries or educational settings. Future research should examine similar psychological predictors across different institutional environments, academic disciplines, and international student groups. Comparative and longitudinal studies would further help clarify how emotional intelligence, cognitive flexibility, and other psychosocial resources interact with institutional and cultural factors to influence academic success and adaptation outcomes over time.

Despite these limitations, the present study contributes to the growing literature on international student adjustment by highlighting the role of emotional and cognitive competencies in shaping both academic performance and broader adaptation outcomes within the Saudi higher education context. The findings suggest that academic success among international students is influenced not only by intellectual ability but also by psychological resources that support effective learning, resilience, and cross-cultural adjustment. Recognizing and supporting these capacities may help universities develop more inclusive educational environments that promote both academic achievement and successful integration for international students.

## 5. Conclusions

This study provides new, important insights into the psychological and sociocultural factors that shape academic success among international students studying in Saudi Arabia. By examining emotional intelligence, cognitive flexibility, language adaptation, and structural characteristics of the student population, the findings highlight the multidimensional nature of academic adjustment in international higher education environments. The study indicates that emotional intelligence is positively associated with academic performance and sociocultural adaptation. Students with stronger emotional competencies appear better equipped to regulate stress, navigate unfamiliar academic systems, and maintain engagement with learning activities. Cognitive flexibility also demonstrated a meaningful relationship with adaptation processes, suggesting that the ability to shift perspectives and adapt thinking plays a critical role in responding to the academic and cultural demands of international study. Together, these findings reinforce the importance of psychological resources in facilitating successful academic integration. Further, the study findings support a multidimensional framework of international student success in which psychological capacities, adaptation processes, and structural conditions interact to influence academic outcomes. Emotional intelligence and cognitive flexibility appear to function as internal resources that help students navigate the challenges of studying abroad, while language proficiency represents a key mechanism through which students translate these capacities into academic achievement.

From a policy and institutional perspective, these findings carry important implications for universities hosting international students. Higher education institutions should recognize that academic success among international students depends not only on academic preparedness but also on emotional and sociocultural adaptation. Universities may therefore benefit from implementing integrated support strategies that address both linguistic and psychological aspects of student adjustment. Language support programs, academic writing workshops, and culturally responsive teaching practices can help students overcome linguistic barriers that may hinder academic engagement. In parallel, programs designed to enhance emotional and social competencies, such as mentoring initiatives, peer-support networks, and wellbeing interventions, may strengthen students’ capacity to cope with the psychological demands of studying in a foreign environment. Institutions should also consider the importance of early adaptation support. Because language proficiency and sociocultural integration appear to improve with time spent in the host environment, targeted interventions during the initial stages of students’ arrival may accelerate the adjustment process and reduce early academic difficulties. Orientation programs that combine language training, academic skills development, and intercultural communication workshops may therefore be particularly effective in supporting international student success.

In conclusion, the present study demonstrates that academic achievement among international students is shaped by a complex interplay of psychological capacities and sociocultural adaptation processes. Emotional intelligence and cognitive flexibility provide important internal resources for navigating the challenges of international study, while language proficiency plays a pivotal role in enabling students to translate these resources into academic success. By recognizing the importance of both psychological and sociocultural dimensions of adaptation, higher education institutions can develop more comprehensive strategies to support international students and foster inclusive learning environments that promote both academic achievement and successful cross-cultural integration.

## Figures and Tables

**Table 1:** Sociodemographic Characteristics of Participants (N = 410).

Characteristic	Category	n (%) or Mean (SD)
Gender	Male	260 (63.4%)
	Female	150 (36.6%)
Region of origin	Middle East & North Africa	178 (43.4%)
	Sub-Saharan Africa	123 (30.0%)
	South Asia	43 (10.5%)
	East/Southeast Asia	23 (5.6%)
	Europe	28 (6.8%)
	Other	15 (3.7%)
Programme level	Undergraduate	143 (34.9%)
	Graduate	267 (65.1%)
Language of instruction	English	169 (41.2%)
	Arabic	211 (51.5%)
	Mixed	30 (7.3%)
Duration in Saudi Arabia	Mean (SD), months	19.8 (11.6)
	<1 year	85 (20.7%)
	1–2 years	230 (56.1%)
	>2 years	95 (23.2%)
Arabic proficiency	Very low (1)	76 (18.5%)
	Low (2)	43 (10.5%)
	Moderate (3)	134 (32.7%)
	Good (4)	123 (30.0%)
	Excellent (5)	34 (8.3%)
GPA (5-point scale)	Mean (SD)	3.64 (0.65)

**Table 2:** Cross-Tabulation of Sociodemographic Variables (N = 410).

**A. Gender × Arabic Proficiency**						
**Gender**	**Arabic Proficiency**	**N**	**% Within Gender**	**Total (Gender)**		
Male	Very Low	40	15.4%	260		
	Low	23	8.8%			
	Moderate	100	38.5%			
	Good	77	29.6%			
	Excellent	20	7.7%			
Female	Very Low	36	24.0%	150		
	Low	20	13.3%			
	Moderate	34	22.7%			
	Good	46	30.7%			
	Excellent	14	9.3%			
**B. Programme Level × Language of Instruction**						
**Programme Level**	**Language**	**Count**	**% Within Programme**	**Total**		
Undergraduate	English	49	34.3%	143		
	Arabic	89	62.2%			
	Mixed	5	3.5%			
Graduate	English	120	44.9%	267		
	Arabic	122	45.7%			
	Mixed	25	9.4%			
**C. Region of Origin × Arabic Proficiency**						
**Region**	**Very Low**	**Low**	**Moderate**	**Good**	**Excellent**	**Total**
MENA	34	26	50	48	20	178
Sub-Saharan Africa (SSA)	20	7	44	44	8	123
South Asia	12	2	10	16	3	43
East/Southeast Asia	4	5	8	5	1	23
Europe	4	3	14	5	2	28
Other	2	0	8	5	0	15
**D. Duration in Saudi Arabia × Arabic Proficiency**						
**Duration**	**Very Low**	**Low**	**Moderate**	**Good**	**Excellent**	**Total**
<1 year	14	12	30	24	5	85
1–2 years	52	20	99	39	20	230
>2 years	10	11	5	60	9	95

**Table 3:** Chi-Square Test.

Comparison	χ^2^	df	*p*	Cramér’s V
Gender × Arabic Proficiency	13.27	4	.010	0.18
Programme × Instruction	11.88	2	.003	0.17
Region × Arabic Proficiency	46.2	20	<.001	0.17
Duration × Arabic Proficiency	82.4	8	<.001	0.32

**Table 4:** Correlations Among Core Study Variables.

Variable	1	2	3	4	5	6
10. Emotional Intelligence (EI)	—					
20. Cognitive Flexibility (CF)	0.54 **	—				
30. Academic Success (AS)	0.48 **	0.31 **	—			
40. Adaptation Outcomes (AO)	0.52 **	0.43 **	0.49 **	—		
50. Arabic Proficiency (AP) †	0.29 **	0.21 **	0.71 **	0.58 **	—	
60. Duration of Residence (DR) †	0.18 *	0.15 *	0.39 **	0.42 **	0.47 **	—

Pearson correlations are reported for continuous variables. Given the ordinal nature of Arabic proficiency and duration of residence categories, Kendall’s tau-b correlations were additionally examined and yielded consistent results. † Arabic Proficiency and Duration of Residence were additionally assessed using Kendall’s tau-b. * *p* < .05, ** *p* < .01.

**Table 5:** Hierarchical Multiple Regression Predicting Academic Success.

Predictor Variables	B	SE B	β	t	*p*	VIF
Block 1: Sociocultural Covariates						
Arabic Proficiency	0.42	0.05	0.48	8.34	<.001	1.82
Duration of Residence	0.16	0.04	0.19	3.87	<.001	1.44
Region of Origin	0.09	0.03	0.11	2.14	.033	1.38
Language of Instruction	0.07	0.04	0.08	1.76	.079	1.25
Block 2: Psychological Predictors						
Emotional Intelligence	0.31	0.06	0.34	5.72	<.001	2.31
Cognitive Flexibility	0.14	0.05	0.16	2.88	.004	2.08

Model Statistics: Block 1: R^2^ = 0.39, F = 18.74, *p* < .001, Block 2: ΔR^2^ = 0.14, Total R^2^ = 0.53, F = 31.62, *p* < .001. Note. VIF = Variance Inflation Factor.

**Table 6:** Hierarchical Multiple Regression Predicting Adaptation Outcomes.

Predictor Variables	B	SE B	β	t	*p*	VIF
Block 1: Sociocultural Covariates						
Arabic Proficiency	0.37	0.05	0.42	7.18	<.001	1.79
Duration of Residence	0.21	0.04	0.24	4.46	<.001	1.46
Region of Origin	0.08	0.03	0.10	2.02	.044	1.39
Language of Instruction	0.05	0.04	0.06	1.42	.156	1.28
Block 2: Psychological Predictors						
Emotional Intelligence	0.39	0.05	0.41	7.06	<.001	2.42
Cognitive Flexibility	0.22	0.05	0.25	4.31	<.001	2.14

Model Statistics: Block 1: R^2^ = 0.34, F = 16.29, *p* < .001, Block 2: ΔR^2^ = 0.19, Total R^2^ = 0.53, F = 34.85, *p* < .001. Note. VIF = Variance Inflation Factor.

**Table 7:** Structural Equation Modeling (SEM) for Structural Path Estimate.

Path	Standardised β	SE	*p*
EI → GPA	0.16	0.04	0.002
CF → GPA	−0.27	0.05	0.001
EI → Adaptation	0.21	0.03	<0.001
CF → Adaptation	0.18	0.04	0.004

Note: Cultural Intelligence (CQ) indicators were collinear with CF; residualised CQ was used to stabilise estimates. Model fit indices were uninformative due to near saturation.

**Table 8:** Mediation Analysis (Bootstrapped) for Indirect Effects (5000 bootstrap resamples). Mediator: Arabic proficiency (proxy sociocultural adaptation).

Effect	Indirect β	95% CI	*p*
EI → Adaptation → GPA	0.026	0.012–0.041	0.001

The indirect pathway was statistically significant, suggesting partial mediation.

## Data Availability

The data presented in this study are available on request from the corresponding author due to ethical restrictions.
